# Letter: Considerations on nonsurgical risk factors for marginal ulcer following Roux-en-Y Gastric bypass for obesity

**DOI:** 10.1097/JS9.0000000000002297

**Published:** 2025-02-07

**Authors:** Qiang Yi, Gangfeng Zhu, Jinghua Zhong

**Affiliations:** a The First Clinical Medical College, Gannan Medical University, Ganzhou, Jiangxi Province, China; b Department of Oncology, The First Affiliated Hospital of Gannan Medical University, Ganzhou, Jiangxi Province, China

*Dear Editor,*

We are profoundly intrigued by the study conducted by Liang *et al*. Their research endeavors to enhance the understanding of identified risk factors, aiming to facilitate more effective preoperative prevention and targeted postoperative interventions for patients undergoing Roux-en-Y gastric bypass (RYGB)^[1]^. The study’s rigorous design offers valuable insights into the prevention of marginal ulcers following RYGB. However, we harbor several concerns regarding the statistical outcomes.

Firstly, the medical subject headings and terminology employed in the search may not sufficiently encompass all relevant literature. Certain studies may utilize alternative terminology to describe marginal ulcers, such as “postoperative ulcers” or “postoperative adverse effects,” while the surgical procedure may be designated as “weight loss surgery” or “metabolic surgery.” This inconsistency in terminology can create blind spots in the literature search, ultimately affecting the overall comprehensiveness of the research conclusions. We recommend that the authors consider employing broader terms and synonyms in their literature screening to ensure a more inclusive capture of relevant studies.

Regarding data integration, while the authors’ prioritization of studies with the largest sample size is indeed a common practice to minimize redundancy and ensure robust statistical power, it may inadvertently exclude smaller studies that offer unique insights or alternative methodologies. These smaller studies could provide complementary or nuanced perspectives that larger studies might overlook, particularly when addressing complex and heterogeneous outcomes. Therefore, while we recognize the validity of prioritizing larger studies, we recommend that the inclusion process should carefully evaluate the quality, relevance, and specific contributions of all studies within the cohort, ensuring that no valuable data or perspectives are omitted. Additionally, when multiple studies cover the same cohort, contacting the original authors to obtain aggregated or supplementary data might help ensure a more comprehensive and balanced integration of findings. By adopting these measures, researchers can better account for the diversity of evidence and enhance the robustness and applicability of their conclusions.

Moreover, the substantial heterogeneity observed among various reported factors in the study, such as marginal ulcers (*I*^2^ = 98.9%), smoking (*I*^2^ = 73.3%), age (*I*^2^ = 67%), alcohol consumption (*I*^2^ = 60.5%), diabetes (*I*^2^ = 64.9%), hypertension (*I*^2^ = 65.5%), NSAIDs (*I*^2^ = 89.5%), and PPIs (*I*^2^ = 94%) indicates significant variability in results among studies. Such high heterogeneity can undermine the credibility of the aggregated conclusions, necessitating a thorough investigation into its sources. A deeper analysis of potential factors contributing to heterogeneity – such as variations in study designs, baseline characteristics of patients, or differing treatment protocols – will enhance our understanding of the applicability and generalizability of these findings.

In conclusion, addressing the aforementioned shortcomings in the conduct of systematic reviews and meta-analyses can significantly enhance the reliability and efficacy of the research. A comprehensive literature assessment, a transparent screening process, and a meticulous quality evaluation are vital steps for bolstering the credibility of the results. Furthermore, researchers should consider establishing unified standards and guidelines to ensure methodological consistency in literature retrieval and data integration. Such standardization not only elevates the quality of research but also provides a reference framework for future studies. We recommend that forthcoming meta-analyses incorporate a broader range of literature and conduct sensitivity analyses to explore the influence of various factors on outcomes, thereby providing more meaningful evidence for clinical practice and ultimately aiding in the postoperative management and prognosis of RYGB patients.

## Data Availability

The datasets are available from the corresponding author on reasonable request.
